# Decoding dye degradation: Microbial remediation of textile industry effluents

**DOI:** 10.1016/j.biotno.2023.10.001

**Published:** 2023-10-26

**Authors:** Soumyajit Das, Lubhan Cherwoo, Ravinder Singh

**Affiliations:** a Department of Biotechnology, Chandigarh University, Punjab, India; b CSIR- Central Scientific Instruments Organisation, Chandigarh, India

**Keywords:** Azo dyes, Bioremediation, Enzymatic activity, Microbial degradation, Phytoremediation

## Abstract

The extensive use of chemical dyes, primarily Azo and anthraquinone dyes, in textiles has resulted in their alarming release into the environment by textile industries. The introduction of heavy metals into these dyes leads to an increase in Biochemical Oxygen Demand (BOD), Chemical Oxygen Demand (COD), and water toxicity. Conventional physicochemical methods for treating textile effluents are costly and energy-intensive. Here introduction of new strategies is eminent, microbial bioremediation for the biodegradation and detoxification of these hazardous dyes, possesses as an innovative solution for the existing problem, discussed are specific groups of bacteria, fungi, and algae which could be one of the potential decolorizing agents that could replace the majority of other expensive processes in textile wastewater treatment by using enzymes like peroxidase, laccase, and azoreductase. These enzymes catalyzes chemical reactions that break down the dye molecules into less harmful substances. Additionally, novel strategies and advancements to enhance the effectiveness of these microbes and their products are comprehensively discussed.

## Highlights

• Synthetic dyes pose environmental challenges due to decolorization difficulty.
• Microbial bioremediation can detoxify hazardous textile dyes.
• Bacteria, fungi, and algae with enzymes decolorize dyes effectively.
• Review emphasizes impacts and strategies for microbial remediation.

## Graphical abstract

**Figure:**
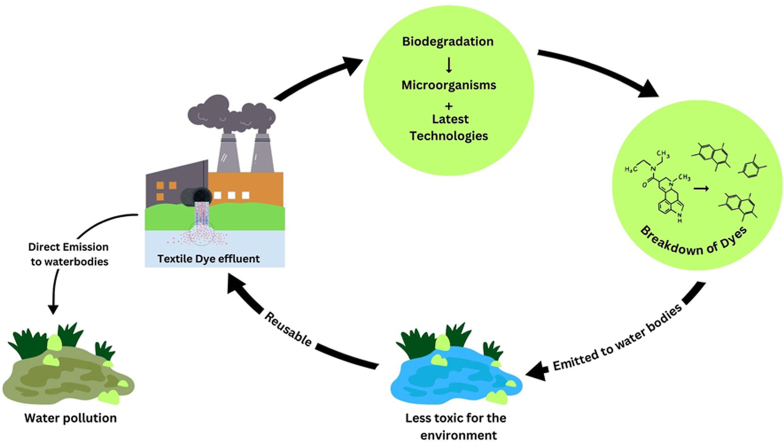

## 1 Introduction

**Fig. 1:**
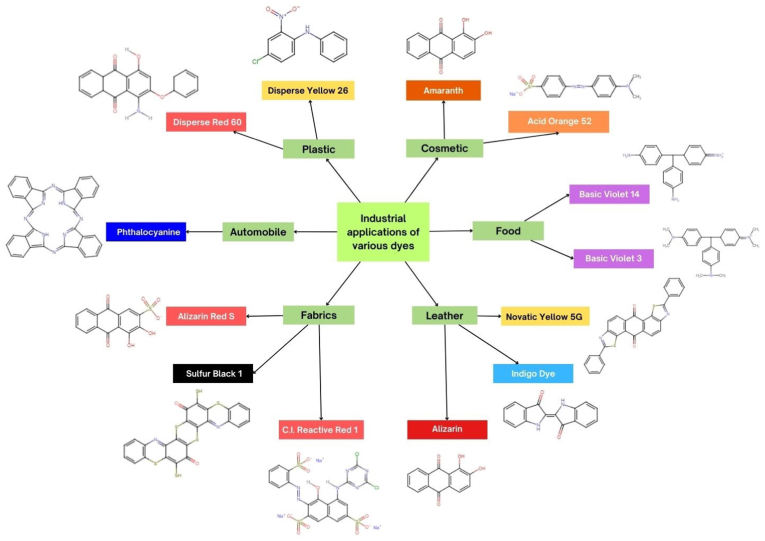
Schematic figure with chemical structure of various dyes used in Industrial process.

Dyes serve as coloring agents employed across multiple sectors, including textiles and paper, to mitigate color susceptibility to interference induced by factors such as washing, heat, light, or otherenvironmental elements that are likely to impact the material.The two components auxochrome andchromophore make up most dyes. The Greek words "auxo" and "chrome" which stand for color and "to increase" respectively, are where the word "auxochrome" originates. Auxochrome is a collection of atoms that can only provide a particular color to a substance when it is joined to a chromophore; on its own, it is unable to do so. The terms "color helpers" and "color intensifiers" are other names for auxochromes. Chromophores are primary components of dyes that absorb and reflect a particular color when subjected to visible light. Based on the chromophore's chemical components, there are about 25 different dye group types available and are used across various industries as colorants, as shown in Fig. 1. A range of features, such as chemical composition, use, and provenance, can be used to classify dyes.

Dyes can be categorized into two categories based on their source, synthetic and natural. Natural dyes are created using resources from nature such as flowers, leaves, and tree bark, whereas synthetic dyes frequently originate through a chemical production process. Prior to 1900, only natural sources were used to make dyes. Synthetic dyes were developed in response to the rising demand and prohibitive costs of extracting natural dyes. Thousands of dyes are used to color a wide range of garments and are categorized as textile dyes. Precursors to dyes are called dye intermediates. With the use of various chemical reactions, they can be produced from basic elements like naphthalene and benzene. Among these, Azo dyes (Procion red, Methylene blue, Congo red, indigo carmine, etc.) are one of the most extensively used and popular in the textile sector. Theseazo dyes are synthetic organic dyes with the functional group RN=NR', in which R and R′ are mainly aryl groups and C–N=N–C linkages.^1^ Some azo dyes are carcinogenic, and some are not. Their carcinogenicity mainly depends on their cleaved products. The second most widely used dye in the textile industry is anthraquinone dyes (Reactive blue 160, Alizarin red, etc.), which contain chromophore groups (C=O).These anthraquinone dyes also have toxic compounds. Metric tons of dye are produced annually, with azo dyes making up about 60–70 % of the total. The main reasons that these toxic anionic dyes are utilized are their effective wet fastness, ease of application, and availability of vibrant color tones. Due to their high molecular weight and the presence of some strong bonds, these molecules are difficult to degrade. The study's relevance and significance lie in addressing the significant environmental challenges related with the increasing usage of dyes, particularly those containing hazardous components. The bioaccumulation of these dyes in aquatic habitats, combined with their potential carcinogenicity, highlights the urgent need for sustainable dye degradation and detoxification solutions. This research seeks to contribute to the development of eco-friendly and efficient dyeing processes, thereby fostering a more sustainable and responsible approach to coloring in many industries by diving further into the mechanisms and techniques for minimizing the environmental impact of dyes.Earlier research suggested 1 kg of the textile product is thought to take 200 L of water and 50–90 g of dye.^2^ Each year, the wastewater from the textile sector releases Approximately 280 thousand tons of dyes used in textiles are released into the environment globally.^3^ Regulations, however, impose a color restriction but do not detail the exact dye restrictions. They merely call for color measurement rather than limits on the amount of coloured water, and do not call for a biotoxicity investigation.^4^ Dye harms the ecosystem even at low amounts. These kinds of commercial textile dyes have chromophore groups that are difficult to degrade. In some situations, these compounds became less vulnerable to the degrading process as a result of an electron deficiency. The type of dyes employed completely determines how many dyes are lost during the dyeing process. The loss will only be 1–2% when using simple dyes, whereas the loss of dyes can approach 50 % when reactive dyes are utilized. Hence, the environment's water quality is negatively impacted by both the method, as it involves discharging dye effluent into water bodies, and the ineffective dying process. Through the physicochemical experiment, it was determined that the BOD and COD levels are by the effect of these toxic dyes.^5^

As a result, along with the aquatic ecosystem, human lives are also heavily affected by this toxic effect.Due to their complicated molecular structures and the need for competent microbial strains to break them down, textile dyes pose considerable environmental concerns.^6^ As a result, intermediate compounds that are dangerous or toxic have been developed, undermining the intended environmental benefits. It has been difficult to scale up lab-size biodegradation techniques to large-scale industrial applications. However, ongoing research is aimed at correcting these flaws in textile dye biodegradation. To improve dye degradation efficiency, researchers are investigating enzymatic techniques, forming microbial consortiums, and investigating nanotechnology. A key field of research is the development of eco-friendly and sustainable dyeing techniques, including non-toxic or natural dyes. Despite these obstacles, recent research shows promise in terms of lowering the environmental effect of the textile sector and promoting sustainable dyeing techniques.

In this paper, a detailed discussion on dyes and their natural impacts is discussed. Although there have been several attempts to study the dye's degradation and a comprehensive work on biodegradation methods, the impacts of textile dyes and the latest advancements in biodegradation are rarely seen. Thus, the novelty of the paper is an overall view of the impact of the dyes, microbial degradation, and knowledge of the specific present and recent developments in treatment for better management of textile wastewater are being made in an effort to protect the environment and raise awareness of the harm that dye-containing textile wastewater causes to natural ecosystems.

## 2 Chemical properties of textile dye effluent

The wastewater from the textile industry is one the major hazardous of all industrial sectors in terms of quantity of output and effluent composition**.**^7^ Sodium hydroxide and other salts are used by the textile industry in wet processing methods for color fixation, resulting in significant salinity and alkalinity (around pH 10–11) in textile effluent. Such textile waste is distinguished by its vibrant color, high biological and chemical oxygen demands, temperature, salinity, pH, salts, and the presence of surfactants and potentially dangerous metals like cadmium, chromium, copper, lead, and zinc, among others. This pollution has an effect on both the environment and human health. In accordance with World Health Organization (WHO) regulations, Table 1 lists several significant features of textile wastewater. However, in most cases, these regulations are not followed properly, as a result, heavy pollution is observed.

**Table 1:** Chemical properties of textile dye effluent as per WHO standard.^8^, ^9^

Parameters	Allowed Range
pH	6.5–8.5
Biochemical oxygen demand (BOD)	150–250(mg L^−1^)
Chemical oxygen demand (COD)	100–300 (mg L^−1^)
Total suspended solids (TSS)	100–00 (mg L^−1^)
Total dissolved solids (TDS)	500–2000 (mg L^−1^)
Chloride	250–1000 (mg L^−1^)
Nitrogen	70–100 (mg L^−1^)
Temperature	Not more than 5 °C above receiving water bodies' ambient temperatures

**Fig. 2:**
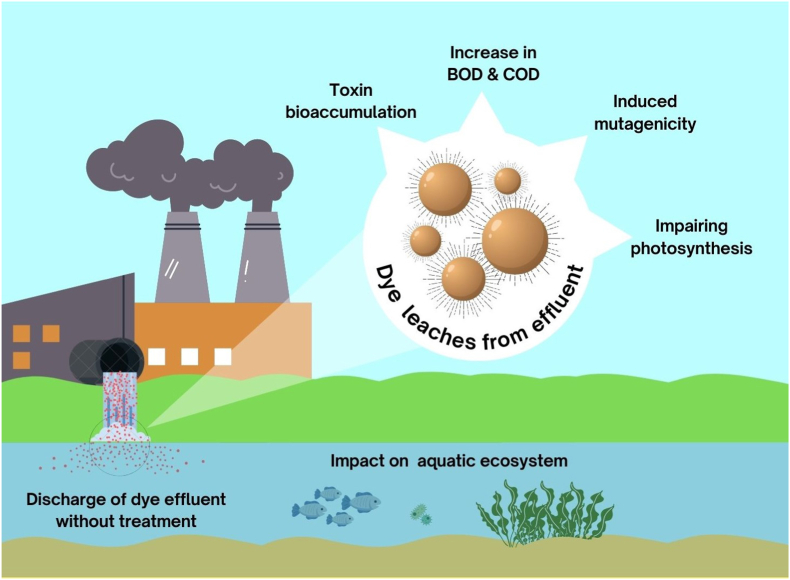
The Eco-toxicological effects of dye-containing textile effluent on the aquatic environment.

The usage of textile dyes may cause a variety of health problems, including bleeding, dermatitis, skin cancer, and perforation of the nasal septum, nausea, ulceration of the skin and mucous membranes, and many respiratory tract irritations.^10^ When inhaled, textile dyes may also cause breathing difficulties, nausea, vomiting, diarrhea, gastritis, and cognitive impairment. Textile dyeing processes often involve the use of various chemicals, including leveling agents, dispersing agents, and fixing agents. The improper disposal of textile dye effluent in bodies of water causes water toxicity which has adverse effects on the surrounding ecosystems as shown in Fig. 2. One of the main effect is severe discoloration of the water, which result in less light reaches the aquatic habitat, which in turn reduces photosynthetic activity. This also leads to anincreased heterotrophic activity, Biochemical Oxygen Demand (BOD) and Chemical Oxygen Demand (COD), which has deleterious influence on aquatic flora and fauna by lowering dissolvable oxygen levels.Several fish species are prone to develop tumors when exposed to these sort of chemicals. It is mutagenic, carcinogenic, and mitotically toxic in severe situations; it can irritate the skin and digestive system, and it has the potential to cause respiratory and renal failure. Many dyes likeprocion red, congo blue, amido black, rhodamine and other such dyes are known to cause eco-toxicity, as explained in Table 2.

**Table 2:** Environmental effect of textile dye effluents.

Dye	Effects on environment
Procion Red^2^	In environment it can be processed to create additional hazardous metabolites, which might result in harmful consequences like allergic responses.
Methylene Blue^11^	If this dye is dumped into the water without any purification, they can seriously harm the aquatic ecosystem because of their low degradative rate and toxicity value.
Congo red^12^	Congo Red had been found to be phytotoxic to aquatic flora and also able to affect the reproduction of aquatic wildlife.
Malachite green^13^	Research shows that malachite green pollution in aquatic environments causes health risks like cancer and respiratory issues.
Anthraquinone^14^	Anthraquinone dyes are extremely difficult to naturally decompose due to their reinforced structure, which poses severe environmental & health issues like endocrine disruption.
Reactive blue 160^15^	Reactive blue 160 dyes hinders sunlight and oxygen supply from reaching aquatic bodies, as a result the oxygen level decreased and toxic level increased in water.
Acid orange^15^	Highly toxic, if entered in the human body it decreased the hemoglobin level.
Amido black^16^	The dye has eco-toxicological effects on the environment. Humans' respiratory systems are harmed by the dye.
Rhodamine^17^	This cationic dye is toxic in nature and may result in negative consequences like skin rashes, dermatitis, and even cancer too.
Methyl Red^18^	Methyl red dye has the capacity of coloration of water, it is poisonous, mutagenic, and produces hazardous byproducts when it undergoes biotransformation.
Crystal Violet^19^	This dye molecule lingers in the environment for a long time and poses environmental hazards. In some fish species, it functions as a mitotic toxin and a powerful clastogene, encouraging the growth of tumors.
Drimarene Blue (Db) K2RL^20^	The dye is toxic, carcinogenic, mutagenic and resistant to degradation

## 3 Classification of textile dyes

**Fig. 3:**
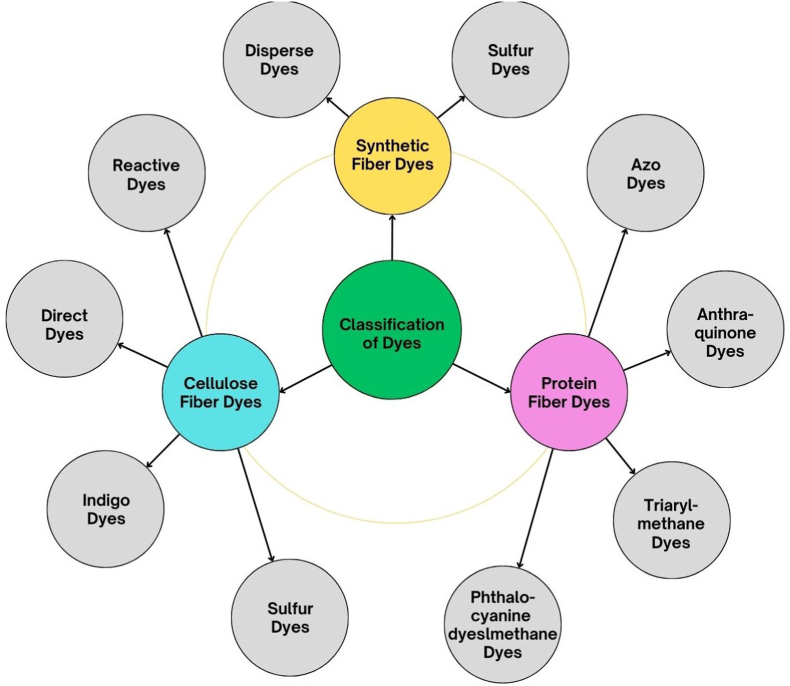
Categories of dyes used in textile and allied industries.

Textile dyes contribute significantly to the brilliant colors and beauty of numerous textile goods. They are categorized depending on their chemical composition, provenance, and manner of application. Based on chemical structure and origin textile dyes can divided into three main categories of cellulose fiber dyes, protein fiber dyes and synthetic dyes, as shown in Fig. 3.

**Fig. 4:**
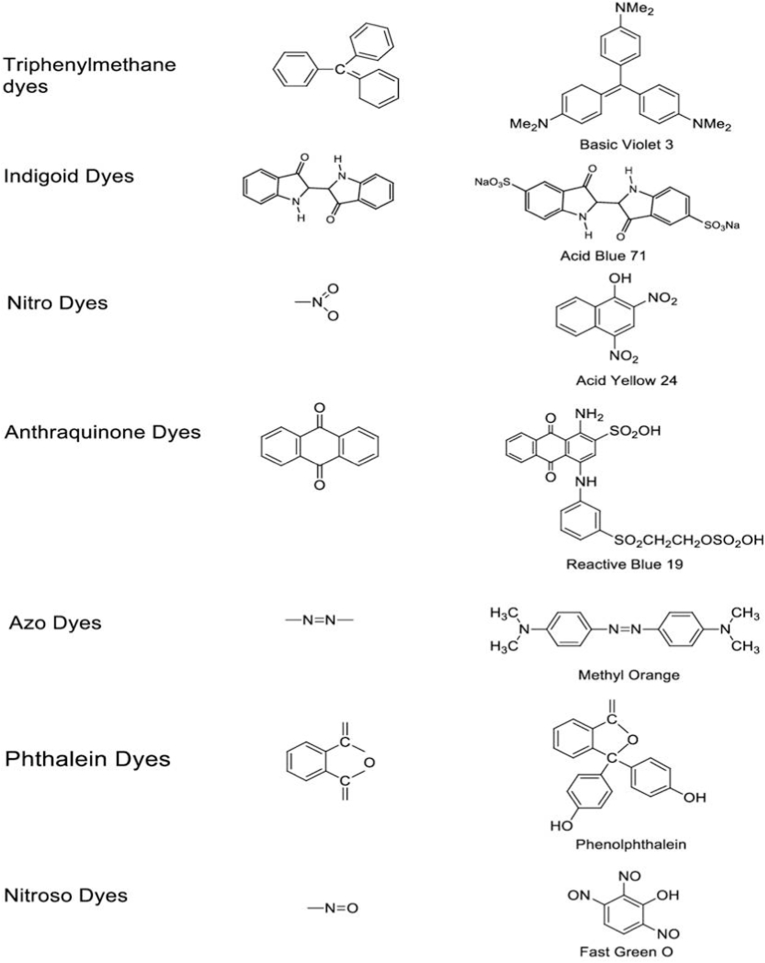
Chemical structures of various types of textile dyes.

The chemical structure of the dyes is one popular categorization, which includes Azo dyes, anthraquinone dyes, nitro dyes, indigoid dyes, phthalein dyes etc. One of the most popular group of dye, Azo dyes are synthetic organic dyes with the functional group RN=NR', whereas another popular group of dye, anthraquinone dyes have chromophore groups (C=O). Another categorization is based on the source of the dyes, with natural colors coming from plants, animals, or minerals distinguished from synthetic dyes manufactured chemically, these can be cellulose fiber or protein dyes, few popular chemical structures are shown in Fig. 4. Understanding textile dye categorization is critical for evaluating their environmental effect, biodegradability, and potential dangers related with their use.

Plants like Flax plant (*Linumusitatissimum*), cotton (*Gossypiumherbaceum*)and ramie (*Boehmerianivea)* are some sources of cellulose fiber. These materials are ideal for reactive dyeing, indigo dyeing, sulfur dyeing, and direct dyeing.^21^ While some protein fibers are responsive to reactive dyes, the majority of cellulose fiber colors fall into this category. They are known for their high pigmentation, long-lasting effect, simplicity of processing across a broad range of temperatures, and adaptability due to the large spectrum of reactive groups that can form covalent bonds with various fibers. Direct dyes, despite their low cost, tend to remain watery rather than adhere to cellulose fibers. To improve their ability to bond the fabric, they are connected with inorganic electrolytes and ionic salts in the form of sodium sulfate (Na_2_SO_4_) or sodium chloride (NaCl).Indigo is classified as a vat dye because it was previously hydrophobic in water but turned hydrophilic after an alkaline treatment. To begin, the water-soluble leuco form of indigo is used to create a precise binding between the dye and the fabric.

Protein fibers used in dyes are derived from animals including silk, angora, mohair, cashmere wool. Due to their susceptibility to high pH value they are dyed with a water soluble acidic dyestuff which deposits insoluble molecules of dyes on fiber. One of the most significant classes of acidic dyes is the azo dyes, which are followed by anthraquinone, triarylmethane, and phthalocyanine. Their versatility, affordability, ease of use, high stability, and strong color make them the major materials of the synthetic dyes market (65–70 %). This color contains a noticeable chromophore (-N=N-) structure, this makes sure the dye is soluble in water and adheres to the fiber.Azo dyes are categorized into three types - mono, bi, and poly (^22^respectively based on how many azo groups are present in their structure.Red dyestuff, in specific, has been utilized for a long time in textile dying sectors where the anthraquinoneclass are well-known for their outstanding fastness, vibrant colors, and water solubility. In the textile industry, triphenylmethane dyes are frequently used to color wool and silk protein fibers. They are made up of two sulfonic acid subunits. If they only include one auxochrome sulfonic acid (So3H) in their chemical structure, they can be employed as indicators. These dyestuffs are widely recognized for their wide color spectrum and water solubility. Because of their light sensitivity, these dyes should only be used on modified polyesters and paper nylon. Cyanine, triarylmethane, anthraquinone, diarylmethane, diazhemicyanine, oxazine, and hemicyanine are their primary chemical components.

Sulfur dyes are relatively small but significant class of dyes because of their exceptional dyeing characteristics, ease of the usage, and affordable cost. Synthetic sulfur dyes are derived from aromatic compounds, such as phenol or naphthalene, which are chemically treated with sulfur compounds to produce the dye molecules. They have a disulfide (S–S) bridge and a complicated structure. They are changed from the keto form to the leuco form using sodium sulfide since they are vat dyes. Water is dissolved with leuco sulfur to generate the coloration. sulfur dyes are not suitable for synthetic fibers like polyester, nylon, or acrylic, as these fibers do not have an affinity for sulfur dyes. Instead, synthetic fibers are typically dyed using other classes of dyes specifically designed for synthetic materials like denim, casual wear, home textiles, and workwear. Dispersed dye is an organic compound that does not have an ionizing component.^22^ They are insoluble in water and are used to color synthetic materials. It is primarily used to color polyester yarn, synthetic fabrics, and is used in acidic environments. Polyester fiber is coloured using disperse dyes, but nylon and acrylic can also be coloured in this manner.

## 4 Methodsof degradation of dyes

**Fig. 5:**
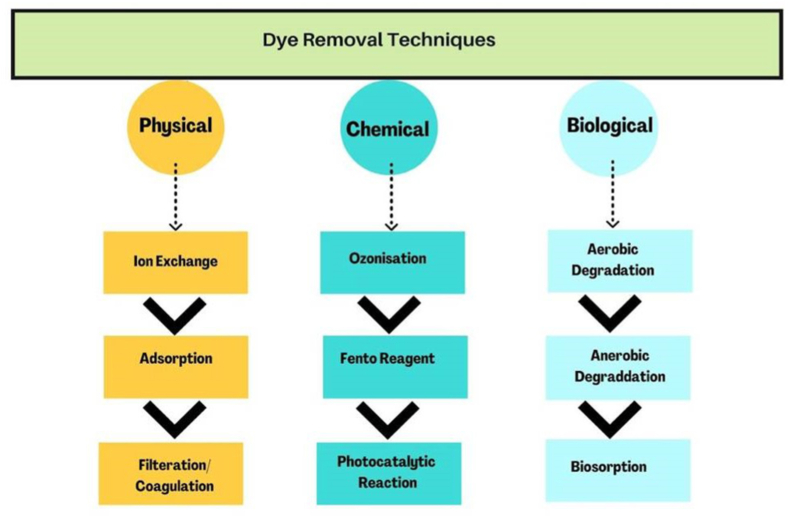
Different types of textile dye degradation methods.

For these previously mentioned reasons, the degradation and detoxification of these dyes are very important. There are various techniques for degrading these dyes: (1) physical methods (i.e., heterogeneous photo catalysis; ii., precipitation; ii., flocculation; iv., ion exchange; v., filtering); (2) Chemical methods (i.e., ozonation; ii., reaction with Fenton reagent); and (3) Biological methods (aerobic and anaerobic degradation of dyes bye the help of microbes). Physical & chemical methods for treating textile effluents are expensive, use more energy, and are not environmentally friendly. Discussions about the development of cheap, eco-friendly methods are currently taking place all around the world. We have focused on the biodegradation methods to degrade dyes which will be advantageous for the environment and probably be a profitable alternative to physicochemical techniques^23^ Fig. 5 shows flowchart for various types of methods employed for dye degradation.

Among these methods, the biological ones that use bacteria are most affordable and effective. In this review paper, we will focus on biological dye degradation methods. Since the last 15 years, a lot of research has been conducted on this biodegradation process. According to these studies, different types of microbes are capable of degrading these toxic dyes. Some examples are *Pseudomonas* sp., *Bacillus* sp., *Saccharomyces* sp., *Streptomyces* sp., *Candida* sp., etc. Due to the presence of some enzymes, these microbes are able to degrade and detoxify the dyes. There are two processes that can be applied for the degradation of textile-emitted toxic dyes: adsorption on microbial biomass or Bio sorption and enzymatic degradation.Biosorption is a surface phenomenon that largely involves the use of surface forces.^1^ We can use bacteria, fungi, and algae for this bio sorption process. The problem with this method is that it does not degrade the toxic dyes into fragments. There are several enzymes present in different microorganisms which help in these degradation processes of textile toxic dyes.

### 4.1 Factors affecting biodegradation of textile dyes

#### 4.1.1 Physical factors

For most of the microbes, the average optimal temperature for their enzymatic activity is around 35–42 °C. The rate of color removal was shown to increase with temperature up to a specific degree, after which there was a modest decrease in decolonization activity. According to some research^24^ after 48 h, the degree of decolonization increased as the temperature rose from 30 °C to 40 °C, and on the other hand reactive black 5's decolonization efficiency decreased to 43 % at the temperature 45 °C. This could happen due to the sudden increase in enzyme activity because of temperature rise up to a certain point. The decolorization activity is greatly diminished at high temperatures (over 55 °C) due to enzyme denaturation. Some textile and other dye waste products are created at relatively high temperatures (50–60 °C) even after a cooling process or heat-exchange treatment.^25^ As a result, the biomass of the utilized living microorganisms must be active and capable of discoloring the effluent at these temperatures, whether alive or dead. The pH of the medium in which this degradation process will be conducted is a very important factor. A high acidic or alkaline pH can slow the rate of dye degradation. The surface charge of microbial biomass is affected by functional groups such as hydroxyl and carboxyl groups. These groupings frequently serve as sites or adsorbing agents. Textile manufacturing activities are usually performed under an alkaline pH, therefore the tolerance level at this high pH is particularly relevant. Therefore, the optimal range for pH is somewhere between 6 and 10. For example, for *Pseudomonas* sp., there are some optimum conditions that increase their dye degradation efficacy, like a pH of 6–10. The lifespan of the bacterial cells and the activity of the azoreductase enzyme are both greatly impacted by the pH value, which lowers the rate of decolonization.^26^^,^^27^Microorganisms cannot utilize toxic azo dyes as their C and N sources.^28^ So these microbial cultures need additional supplementation from these sources in the media. The majority of dyes have low carbon levels. This makes the microorganism-based color removal procedure challenging in the absence of an additional carbon source. Carbon sources such as glucose and starch boosted bacterial growth and metabolism, increasing the percentage of dye decolonization. The chemical composition of the dye and the microbes participating in the process of decolonization may influence the difference in nutritional requirements. According to,^29^ *Bacillus endophyticus* LWIS1 used yeast extract as a carbon source and degraded 98 % of the Remazol black-B after 10 h of incubation. It demonstrates that yeast extract functions as a co-substrate and boosts the decolonization efficiency of *B. endophyticus LWIS1*. Nitrogenous supplements included ammonium sulfate, ammonium chloride, ammonium nitrate, and peptone among the nitrogen sources used in the decolonization study of dyes.

#### *4.1.2* Chemical factors

One of the key components in the growth of microbial cells is oxygen. There are different kinds of microbes, like some that are aerobic, some that are anaerobic, and some that are facultative in nature, so their oxygen demands are different. However, in some circumstances, the dye reduction rate slows if the extracellular environment is aerobic because of the presence of the oxygen molecule (a high-redox potential electron acceptor).^30^*Bacillus* sp. *strain CH12* successfully decolorized the reactive red 239 dye about 90–100 % under anoxic and anaerobic conditions, but the decolonization percent was only 18.6 % when it was shaken.^31^As a long-term electron acceptor, oxygen is superior to azo groups, and dissolved oxygen often inhibits the anaerobic decolonization of azodyes. Increasing the amount of a dye's toxicity could lower the rate, efficiency, and survival of microbial decolonization.^32^^,^^33^ The relationship between the dye concentration in a solution and the readily available sites on an adsorbent surface determines the impact of the initial dye level in the bioremediation process. Changes in dye molecular structure may be responsible for the variance in decolonization percentages; dyes with a less complex structure and a lesser molecular weight decolorize more quickly than those with a more intricate structure and greater molecular weight.

## 5 Microbial degradation of textile dyes

**Fig. 6:**
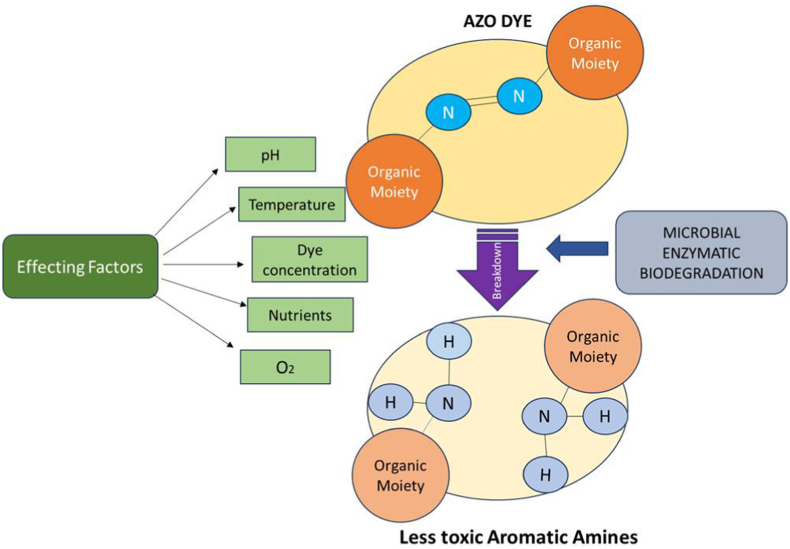
Mechanism for microbial biodegradation of dyes.

Numerous adaptable microorganisms, including bacteria, fungi and algae which are present in wastewater and/or polluted environments, are responsible for the biodegradation and bioremediation processes, as shown in Fig. 6. This method might also be carried out in a laboratory setting by selecting and isolating the suitable microbes, followed by a scale-up that allows for the treatment and decolonization of textile effluents. Microorganisms in charge of biodegradation may use a variety of methods, such as adsorption, bio sorption, alleviation, bioaccumulation elimination, or conversion of toxic waste molecules into harmless or even beneficial compounds. Procion Red is one of the widely used azo dyes in industry*.* Due to their high molecular weight and the presence of some strong bonds (Azo bonds), these molecules are difficult to degrade. According to research, *Pseudomonas stutzeri SPM-1* can degrade this protein. Pseudomonas is a gram-negative, rod-shaped, motile bacteria. It is a facultative aerobic bacterium. The data shows that the enzyme activity of azoreductase and NADH-DCIP reductase present in this Pseudomonas species is the reason behind this degradation.^2^

There are some optimal parameters like pH around 6–10 and temperature around 35 °C increases the efficiency of dye degradation by Pseudomonas sp. The rate of decolonization increased when carbon and nitrogen sources were utilized as co-substrates. *Saccharomyces cerevisiae* is a unicellular fungus. Saccharomyces cerevisiae cells bioaccumulatediazo-reactive textile dyes (Remazol Blue Dye and Remazol Black B Dye) when growing in molasses. The hazardous azo dye methyl is red and is discolored by Saccharomyces cerevisiae MTCC 463 in a static anoxic environment at around 35–40 °C.^34^ This breakdown process is driven by the enzymes laccase, azoreductase and NADH-DCIP reductase. After being decolored in ordinary distilled water, the cells' lignin peroxidase and NADH-DCIP reductase activity are considerably enhanced. Wastewater from the textile industry can be treated with Saccharomyces species, which are non-pathogenic, convenient, and affordable biomass. Anion exchange and molecular sieve chromatography were used to separate Pseudomonas sp. SUK1's internal bacterial peroxidase from other bacteria. The purified enzyme's potential role in the decolonization of dyes was assessed*.* Due to its peroxidase activity, this strain of *Pseudomonas* detoxifies the carcinogenic anthraquinone dyes.^35^ It was discovered that the enzyme's substrate specificity had changed. Peroxidase is an effective enzyme for the detoxification of dye wastes due to its capacity to decolorize various textile dyes.

Methyl orange undergoes symmetrical cleavage by peroxidase, yielding 1,4-benzenediamine, N, N-dimethyl, 4-aminobenzenesulfonic acid, and aniline. *Phormidiumautumnale*is a cyanobacterium present in soil and freshwater.*Phormidiumautumnale* Degrade the indigo dye due to the production of metabolites like anthrallic acid and isatin.^36^ In terms of evaluations for discoloration and toxicity, the cyanobacteria's capacity for dye breakdown was compared to that of anaerobic and anaerobic-aerobic systems. By using absorption spectroscopy, the discoloration was assessed. The*Selenastrum capricornutum* alga, lettuce seeds, and the organism *Hydra attenuata* were used to test for toxicity. The indigo pigment could only be fully destroyed by *P. autumnale UTEX 1580*, a single strain of cyanobacteria. The micronucleus assay was employed on *Allium* sp. to examine the potential for mutagenicity. After the treatment, there was no evidence of mutagenicity. *Candida albicans* is pathogenic yeast. Numerous studies using *C. albicans* show that yeast has the ability to produce reductase, which may be used to break down a variety of substrates, including dyes*.* Direct Violet 51 is a carcinogenic dye. Due to the production of primary and secondary amines (metabolites produced by *Candida* sp.), the dye is degraded into nontoxic compounds.^37^ The removal of the pigmented substance by the yeast *C. albicans* showed a possible enzymatic ability to change the chemical structure of this dye, which is frequently found in industrial effluents. The potential of a *Neurosporacrassa* fungal bodies to produce oxidative enzymes and their utilization in the biodegradation of phenolic compounds was demonstrated in static and shaken non-immobilized batch cultures, along with capillary membrane-immobilized biofilms. *Neurosporacrassa,* commonly known as pink bread mould, is a filamentous ascomycete which is able to produce two different oxidoreductases. A polyphenol oxidase and a laccase, in particular, inhibit the activity of the dyes.^38^The immobilization of *N. crassa* biofilms enables the production of oxidase enzymes continuously, which has been proven to be an effective method for the conversion of phenol and p-cresol, two hazardous phenolic wastes. A triphenylmethane dye called malachite green is utilized in the textile sectors.It is also harmful to life forms, according to reports. *Streptomyces chrestomyceticus* is a spore-forming gram-positive bacteria. Malachite green is degraded into a nontoxic compound due to the activity of the Azoreductase enzyme present in this species.^39^

The toxicity experiments showed that the metabolites of malachite green breakdown by *Saccharomyces chrestomyceticus S20* were less hazardous to human cells than the parent MG (Malachite green) and neither toxic to plants nor microorganisms. This is the study on the biodegradation of MG(Malachite green) by *Saccharomyces chrestomyceticus*, a prospective candidate to remove MG from diverse areas. Significant use of the Rhodamine-B dye has been made in every industry and branch of science. It is harmful to human organs and mutagenic. Utilizing a brand-new bacterial strain that efficiently falls down the Rhodamine-B dye According to some research, the bacterial strain *Brevundimonasdiminuta* is capable of degrading the color Rhodamine.^40^ The unique aspect of the research is how well this strain works with rhodamine dye degradation. This degradation examination is characterized by a UV–visible spectroscopy analysis, and the metabolites are subsequently studied by GC-MS and FT-IR analysis.^41^ Utilizing endophytes with superior dye degradation potentials could be more advantageous than bioremediation by free-living microorganisms. The enzyme-mediated hydrolysis of a precursor named ACC deaminasehas also revealed endophytes that help plants recuperate from the stress brought on by textile effluents.^42^ Unexpectedly, eight out of the forty-one endophytes from the bacterial genera of *Bacillus*, *Microbacterium*, and *Halomonas* not only shown the best efficiency in dye degradation but also promoted plant growth by generating siderophore, IAA, phosphate solubilization, and ACC deaminase activity. The dispersed blue 284 dye was decolored using the potential microbiological tool *Klebsiellapneumoniae* GM-04 in a current bio remedial approach.^43^ From industrial effluent, *Klebsiellapneumoniae* was isolated. In 24 h, *Klebsiellapneumoniae* resulted in a 90 % decolonization of DB-284 under ideal circumstances (37 °C and a pH of about 7). Low levels of dye discoloration were caused by a combination of a temperature reduction and an increase in dye concentration. Numerous bacterial enzymes, such as reductases, laccases, and oxygenases, have the ability to break down azo linkages through reductive degradation under anaerobic conditions and then biologically convert aromatic amines under aerobic conditions. A significant amount of effluents containing various harmful colors is produced by the textile sector. Indigo, carmine, and malachite green are the most frequently used dyes in the textile and dye industries. An alkali-thermostablelaccase is produced by Bacillus subtilis DS.^44^

The conditions for the immobilization of this enzyme alkali-thermostable laccase is produced by Bacillus subtilis DS were optimized, and it was immobilized on chitosan beads. Numerous techniques for immobilizing laccase have been thoroughly investigated, such as adsorption, covalent binding, entrapment, and crosslinking. Entrapment stands out as a favorable option due to its gentle procedure, which minimizes disruption to the enzyme's native structure**.**^45^Textile colors were degraded utilizing immobilized laccase, and the settings were statistically standardized for the best degradation. Under ideal conditions, high degradation rates of indigo carmine (95 %) and malachite green (90 %), respectively, were achieved. Microbial biosorption is a technique that makes use of microorganisms' capability to bind to effluents and remove contaminants from polluted soil or water, including textile dyes. The microorganisms, such as bacteria or fungi, possess functional groups on their cell surfaces that can interact with dye molecules, allowing for their adsorption and subsequent removal. The bioremoval capability of *Platanusorientalis* tissue from the leaves (NSPOL) prepared utilizing a passive immobilization approach was examined in the study. Under varied experimental conditions, its efficiency in removing reactive red 198 or RR198 and reactive yellow 2 or RY2 dyes was carefully tested.^46^ The study's findings show that NSPOL has a great performance in effectively removing various dyes, highlighting its promising potential for dye remediation. Table 3 highlights various studied and methods involved in microbial degradation of textile dyes.

**Table 3:** Microorganisms involved in dye degradation.

Microorganisms	Dye	Efficiency
Brevundimonasdiminuta^40^	Rhodamine-B	90–95 %
Pseudomonas stutzeri SPM – 1^2^	Procion red	90 %
Streptomyces chrestomyceticusS20^39^	Malachite green	96 %
KlebsiellaPneumoniae GM-04^43^	Disperse blue-284	90 %
Phormidium autumnale^36^	Indigo dye	60 %
Saccharomyces cerevisiae ATCC 9763^47^	Methyl red	75 %
Pseudomonas sp. SUK1^35^	Anthraquinone Dyes	80 %
Candida albicans^37^	Direct violet 51	73–87 %
Bacillus subtilis ‘RA29’^48^	Rhodamine, acid orange	98.23 % decolonization of Congo red, 78.32 % Amido black, 96.69 % Acid orange
Aeromonas sp.^49^	Methyl Orange	99 %
Providencia sp. SDS^50^	Remazol Black, Red HE8B	84 %
Bacillus pseudomycoides^44^	Acid Black 24	96 %
Acinetobactercalcoaceticus^51^	MR,MO	90–98 %
Sulfate-reducingbacteria(SRB)^52^	Orange II	95 %
Staphylococcus sp.^53^	Reactive Blue	97 %
SterigmatomyceHalophilus SSA1575^54^	RB-5	100 %
Bacillus cereus SKB12^55^	RB-5	88.7 %
Aspergillus Foetidus^56^	Azo Reactive dyes, Drimarene	95 %
Chlorella vulgaris^8^	MB and AO7	83 %
Enterobactersp.CV–S1^57^	Crystal Violet	100 %
Enterococcus Faecalis R1107^58^	CongoRed	90.7 %
ShewanellahaliotisRDB_1^59^	Reactivered120	100 %
Bacillus sp. strainCH12^31^	Reactive Red 239	100 %
BacillussubtilisMTCC 441^60^	Methylene Blue	91.6 %
Pseudomonas Putida^61^	ReactiveYellow 174	90 %
Synechococcus Elongatus^62^	Malachite Green	99.5 %

## 6 Overview of microbial enzymes

**Fig. 7:**
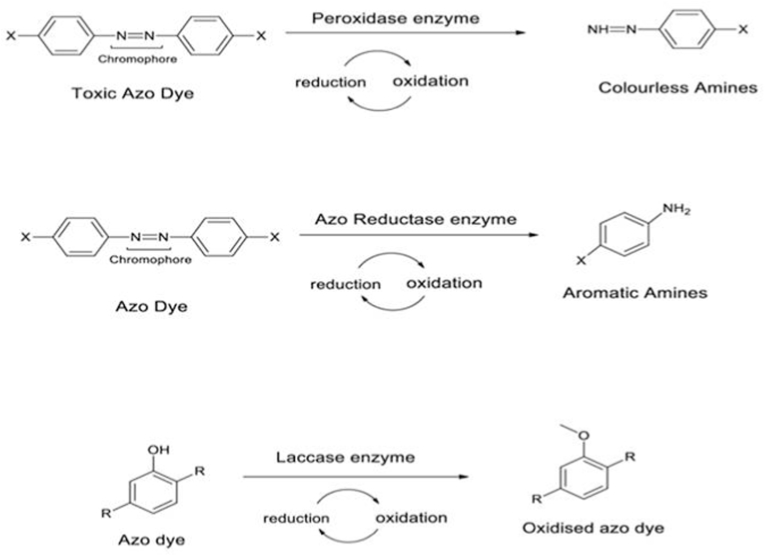
Microbial enzymes structures and reaction.

Microbial enzymes, such as peroxidase, azoreductase, andlaccase, in the biodegradation of synthetic dyes are prominently used in biodegradation of components of textile effluents, these enzymes offer promising alternatives to conventional methods for dye removal due to their ability to efficiently break down complex dye moleculesas shown in Fig. 7. Peroxidase exhibits broad substrate specificity and mild reaction conditions, while

azoreductase specializes in the degradation of azo dyes. Laccase, on the other hand, is effective in breaking down phenolic compounds. Understanding the enzymatic mechanisms involved in dye degradation can contribute to the development of solutions for textile wastewater treatment.

The oxidoreductase enzyme family (peroxidase, NADH-DCIP reductase, oxygenase, oxidase, etc.) is called an oxidoreductase because they facilitate the movement of electrons from one type of molecule—such as an oxidant, an electron donor—to another, the reductant, an electron acceptor. They need NAD(P), FAD, and FMN as cofactors for the reduction process.^63^ Contrary to laccases, peroxidases use a similardye degradation mechanism to oxidize their substrates (azo dye molecules) by using H_2_O_2_ as their electronterminal receptor. The two most widely employed ligninolytic peroxidases for color degradation are lignin peroxidase and manganese peroxidase. Lignin peroxidase and manganese peroxidase, both derived from the fungus *P. chrysosporium*, have demonstrated in vitro the effect of high-redox potential lignin peroxide in dye decolonization using pure enzymes.^64^However, many oxidoreductases perform best at neutral pH (about pH 7).The temperature optima for oxidoreductase activity generally range between 20 and 40 °C, but this can vary depending on the enzyme and organism. The combination of various enzymes, including laccase, azoreductase, oxidoreductase, and others, can significantly enhance the biodegradation of textile dyes. It's important to notethat the choice of enzyme combination and the specific enzymes used depend on the dye type, its chemical structure, and the microbial species involved.

Azoreductase is part of the flavin-containing family of enzymes.Azoreductases are typically produced by bacteria and fungi and are involved in the reduction of the azo bonds This important reductive enzyme azoreductase exclusively catalyzes the redox-mediated reductive cleavage mechanism via which endophyticbacteria can degrade textile azo colors.Azo dye reductases are classified as membrane-bound or cytoplasmic, and for the degradation of azo dyes, they both primarily use reducing equivalents like NADPH, NADH, and FADH as catalysts. They are one of the most important enzyme groups involved in the breakdown of textile dyes. They do this by cleaving the azo bond present in these dyes, which produces less toxic aromatic amines.^42^The optimum pH for azoreductase activity usually falls within the range of 6–8, although some enzymes may exhibit optimal activity at slightly acidic or alkaline pH values. The temperature optima for azoreductase activity typically range between 25 and 40 °C, but this can vary depending on the specific enzyme and organism. Some reducing agents, such as FADH2, NADPH, and NADH, were required for this reaction. Under anaerobic conditions, the membrane-bound azoreductase enzyme breaks the N=N double bond of the colorful hazardous dye molecules by transferring electrons to the electron acceptor, azo dye, and transforming them into colorless aromatic amines that are nonetheless poisonous. Under aerobic or anoxic circumstances, these hazardous amines further mineralize into non-toxic molecules.

Laccase enzymes are from the oxidase family and have multicopper incorporated into their structure.^65^ A wide range of textile dyes, including azo dyes, anthraquinone dyes, andtriphenylmethane dyes, can be reacted with by laccases because to their broad specificity for the substrate.Byreacting on the azodye phenolic group via a free radical mechanism, they can non-specifically catalyze the breakdown of different azo dyes, making phenolic compounds and producing less toxic aromatic amine byproducts. They initially catalyze the asymmetric breakdown of the N=N double bond of their molecule in the process of degrading azo dyes, which is subsequently followed by oxidative cleavage, desulfonation, deamination, and dehydroxylation. It has been observed that some bacterial laccases break down azo dyes by producing phenolic chemicals as opposed to cleaving the azo link through a very ambiguous free radical mechanism. The optimum pH for laccase activity generally ranges between 4 and 7, depending on the specific laccase and the dye being targeted. However, certain laccases have been reported to function optimally at higher pH values, up to pH 9. The temperature optima for laccase activity typically fall within the range of 25–60 °C, although some laccases can remain active at higher temperatures.A combination of crude laccase and copper iodide nanoparticles achieved rapid degradation of mixed azo dyes, outperforming other degradation methods, with in silico analysis predicting strong enzyme-dye interactions.^65^

7. Present development and new advancements in microbial biodegradation of textile dye effluents.

**Fig. 8:**
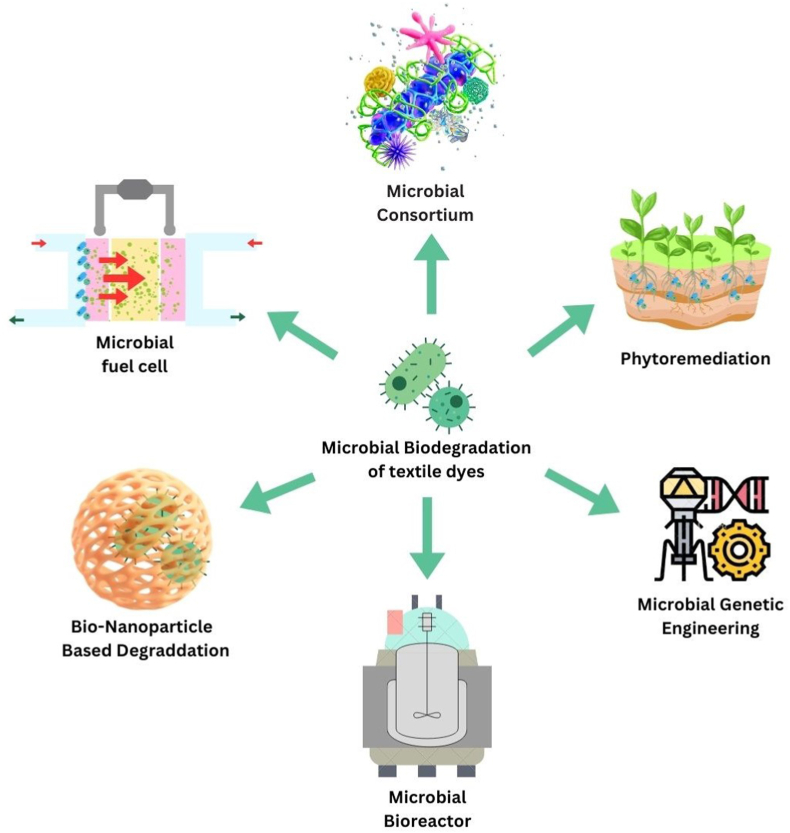
Different Combinatorial Treatments of textile dyes.

Significant progress has been made in the field of biodegradation for the treatment of textile effluent dyes, in recent years microbial biodegradation has emerged as a viable and long-term solution for reducing the environmental effect of textile dye pollution. With increasing demand in better alternatives and sustainable techniques to treat effluent dyes in textile waste water it is eminent to study and develop new methods. One such method is the utilization of microbial consortiums and genetically altered microorganisms, which has shown promise in improving degrading efficiency. To increase the efficacy and stability of microbial degradation systems, advanced approaches such as enzyme immobilization, biofilm development, and the use of nanomaterial has also been studied. To achieve more effective and complete textile dye wastewater treatment, the combination of microbial biodegradation with other physicochemical treatment approaches has also been investigated, as depicted in Fig. 8. These recent advances in microbial biodegradation show considerable promise for long-term and environmentally beneficial solutions to the difficulties faced by textile effluent dyes, including analyzing their environmental effect, biodegradability, and possible dangers connected with their usage.

### 6.1 Microbial consortium

A microbial consortium is typically described as a collection of several microorganisms with the capacity to cooperate in a community. There are some advantages to using these microbial consortiums rather than a single species of microbes. For example, each species can act on a different target molecule and can be consumed or detoxify various products produced as a result of the dye degradation Different members of a microbial consortium possess a wide range of enzymatic activities, including oxidases, reductases, hydrolases, and decarboxylases. These enzymes have the capability to degrade various types of dyes by breaking down their complex chemical structures. This results in a synergistic action where the presence of additional microorganisms positively affects the enzymatic activity of another microbial species, boosting the rate of breakdown. For example, study demonstrates that a combination culture of fungi named Penicillium sp. QQ and a bacterial strain named *Exiguo bacterium* sp. TL can work synergistically to breakdown the textile dye more efficiently. K–R Reactive Dark Blue.^66^*Marinobacterium* colonized the native microbial population and positioned itself as the dominant bacterial genus in the bioaugmented HA, where it performed an important role in azo dye decolonization^67^ As a result, bioaugmentation with a halophilic bacterial consortia improved the HA system for azo drug decolonization.

### 6.2 Microbial fuel cells

Microbial fuel cells, or MFCs, are a potential bio-electrochemical technology that can produce electricity while reducing organic and inorganic pollutants. As catalysts, MFCs oxidize organic compounds.^68^ As organic compounds oxidize, electrons and protons are produced at the anode and travel to the cathode via an external circuit to complete the electrochemical reaction and generate energy. Electron transfer is required for dye decolonization because some of the produced electrons are contributed to the dye for reductive decolonization while others are carried to the anode for electricity production.According to research, an integrated single chamber microbial fuel cell (SMFC)-aerobic bioreactor setup efficiently decomposed azo dye (Acid Blue 29) into less contaminated biodegraded products utilizing acetate as a co-substrate.^69^When microorganisms in the MFC degrade organic matter as a fuel source, they generate metabolic byproducts and intermediate compounds. Some of these byproducts or metabolites can have an indirect impact on dye degradation. The application of microbial fuel cells in textile dye biodegradation shows promising potential for sustainable and efficient treatment of textile dye wastewater, contributing to a cleaner environment. Ongoing research in this field aims to further improve MFC technology for effective dye degradation and commercial viability.

### 6.3 Phytoremediation

Phytoremediation is an environmentally friendly and cost-effective process that reduces the toxicity of pollutants through the combined action of some plants and their root-associated microbes. This is an example of a synergistic relationship where both species act together for a better result. Textile dye phytoremediation is a new and promising approach. Recent research has found that phytoremediation of textile dyes by *Boutelouadactyloides* and three species of bacteria (*Ochrobactrum* sp.*, Pseudomonas aeruginosa, and Providenciavermicola*) reduces the toxicity level of textile-emitted effluent dyes after the treatment.^70^ The combined actions of the plant's phytodegradation, phytoaccumulation, and the enzymatic action of bacteria drive the whole remediation process. Phytoremediation is a promising approach that is economically viable and ecologically sustainable. Microbial phytoremediation offers several advantages, such as low cost, sustainability, and the potential to treat large areas of contaminated soil or water. However, the success of microbial Phytoremediation for textile dye removal depends on various factors, including the choice of suitable plant species, the presence of specific dye-degrading microorganisms, and optimization of environmental conditions.

### 6.4 Nanoparticles-based bioremediation

The hazardous Methylene Blue dye was removed from artificial aqueous solutions through batch adsorption tests employing the nanoparticle form of Arthrospira platens (NIOF17/003.^71^; Green nanotechnology is a term used to describe a process for making nanomaterial's that is devoid of or employs less toxic ingredients. The system containing the textile dyes is perturbed by the nanoparticles. Large-surface-area nanomaterials are a great option for the treatment of wastewater containing dyes because they have special electron conducting capabilities that change their surface properties. Microorganisms, such as bacteria or fungi, are either naturally present or introduced into the system. The nanoparticles interact with the microorganisms, forming a synergistic partnership for dye degradation. Green synthetic NPs can be produced using a variety of biological components, including bacteria, actinomycetes, fungi, macroalgae, and plants. Green synthesis is preferred over chemical and physical methods due to its sustainable development, affordability, simplicity of handling, upscaling, and biological compatibility.

### 6.5 Genetic engineering of microbes

The creation of recombinant bacteria and plant species with genes encoding excellent dye-degrading enzymes is another important and fascinating field of study. By using proper genetic manipulation, we can achieve our desired result in the degradation process. Many studies have been conducted to identify transgenic bacteria in ecological systems and to generate genetically altered microorganisms that are suicidal to prevent the spread of these bacteria into naturally occurring microorganisms. Some of the most recent studies have reported the use of various immobilized microbial strains for decolonization. Dyes like Reactive Black 5, Alizarin Red S, and Remazol Brilliant Blue R were eliminated using *TrametesVersicolor* fungal strain immobilized on oxide materials of Tio2-Zro2-Sio2 (molar ratio of 8:2) and Tio2-Zro2-Sio2 (molar ratio of 8:1:1), which were used under the best process conditions of pH 5 and 25 °C at an initial dye concentration of 90 %.^8^

### 6.6 Role of bioreactors

The establishment of high-performance efficient bioreactors is critical for the effective use of textile dye treatment in industrial effluent.Bioremediation is presently one of the major promising options for treating dye-containing synthetic waste that contains harmful dyes. The most popular device, the bioreactor, has been widely employed to carry out the most challenging biological reactions. It can work continuously or in batches. Membrane bioreactors, fluidized beds, moving beds, packed beds, airlift bioreactors, stirred tank bioreactors, fixed beds, and rotating biological contactors have all received special attention.^72^ In a modified rotating packed disc bioreactor (RPDB), mixed cultures of white-rot fungus (WRF) are utilized to treat synthetic reactive black 5 wastewater. Using the mixed fungal culture, the dye effluent was entirely decolored with a chemical oxygen demand reduction of more than 93 %.^73^ By holding the biomass for a long time, these reactors produce high reaction kinetics per unit reactor capacity. Reduces the toxicity of the waste and reduces the cost of leachate disposal produced during the process. These reactors control all the parameters (pH, temperature, oxygen concentration, level of agitation, etc.) efficiently.

## 7 Future of microbial degradation of textile dyes

Biological treatment of textile industry effluents faces a number of difficulties despite its potential. The complexity and variety of dye compounds, the toxicity of some dyes, and the existence of inhibitors are a few of these. Further research is needed in the areas of bioreactor efficiency and the adaptability of microbes to the various effluent compositions. More research is needed in order to get effective outcomes from dye biodegradation. A number of crucial issues must be resolved like.^74^ A) Dyes with a high molecular weight and complex structure pose challenges in degradation processes. Their breakdown requires specialized approaches and further investigation. B) Degradation of different types of dyes requires more than one strain of microorganism. Different dyes may require distinct microbial strains or combinations thereof. Therefore, the selection of appropriate strains or microbial consortia needs exploration. C) It is essential to take into account the toxicity of the by-products produced during the degradation process. Studying the impact of some of these byproducts is important since they might have negative environmental repercussions. D) Maintaining optimal enzyme activity requires careful control of additional supplements and parameters. The identification and optimization of these factors are critical for achieving efficient dye biodegradation.

More research is needed to identify bacteria capable of breaking down and mineralizing combinations of colors since actual textile dye effluent contains multiple hues. Instead of relying solely on model pollutants used in laboratory settings, genuine textile industry effluent should be employed for a more accurate assessment of how isolated strains would perform in practical industrial applications. The removal or recovery of bacterial isolates following investigations on dye degradation is another issue that needs to be addressed. Currently, there is a lack of literature focusing on the fate of these bacterial isolates. Future studies should determine the possibility of recovering these isolates after dye degradation is complete. Dye degradation processes can generate valuable byproducts, such as food and energy sources, which can be traded as commodities. However, achieving complete dye degradation remains a challenging obstacle that researchers need to overcome. For effective application of dye wastewater biodegradation, numerous scientific trials must be conducted. Future research should focus on identifying inhibitors of microbial activity to improve the efficacy of recent and promising experiments. Bioremediation has been transformed by omics methods, such as meta-genomics, meta-transcriptomics, meta-proteomics, and metabolomics, which have deep insights into the mechanisms and key enzymes involved in pollutant breakdown.^75^ By improving our knowledge of the dynamics and activities of microbial populations in disturbed environments, these technologies enable more efficient and environmentally friendly bioremediation procedures. Governmental organizations like the Environmental Protection Agency (EPA) advocate the integration of omics approaches because it provides a thorough understanding of intrinsic microbial communities, their behavior, and their functional potential in pollution containment systems.In conclusion, biodegradation offers several advantages over traditional techniques, including being non-hazardous, environmentally friendly, cost-effective, and generating less waste. However, further research is essential to enhance the efficiency and applicability of biodegradation processes.

## 8 Conclusions

Textile effluent, especially that containing toxic dyes, must be properly treated before it is released into the environment. This situation has serious consequences for the polluted area when toxic textile dyes are ejected into the water without treatment. such as interfering with the entry of sunlight into the water, impacting photosynthetic organism's metabolism, decreasing the water's O_2_ level, and affecting the normal activity of flora and fauna. This can be toxic for animals and humans if ingested or acquired through skin contact. Biotechnology has been extensively used in the quest for dye degradation, primarily because biological alternatives are more efficient and have a lower environmental impact. The currently used bioremediation techniques are mostly laboratory-scale. One of the biggest technological challenges is integrating these technologies for use in extensive commercial applications. The kind of effluent, the toxicology of the metabolites, the accompanying expenses, and the intended application of the water that has been treated are all factors to consider when adopting bioremediation. On the basis of available scientific research, we discussed the biodegradation of textile dyes in this review. To obtain optimum decolorization by the isolated microbial strains, all parameters must be optimized. Furthermore, with recent developments in omics studies and nanotechnology development, it is possible to improve the performance of microbial enzyme activity in textile toxic dye treatments.

## Author declaration template

We wish to confirm that there are no known conflicts of interest associated with this publication and there has been no significant financial support for this work that could have influenced its outcome.

We confirm that the manuscript has been read and approved by all named authors and that there are no other persons who satisfied the criteria for authorship but are not listed. We further confirm that the order of authors listed in the manuscript has been approved by all of us.

We confirm that we have given due consideration to the protection of intellectual property associated with this work and that there are no impediments to publication, including the timing of publication, with respect to intellectual property. In so doing we confirm that we have followed the regulations of our institutions concerning intellectual property.

We understand that the Corresponding Author is the sole contact for the Editorial process (including Editorial Manager and direct communications with the office). He/she is responsible for communicating with the other authors about progress, submissions of revisions and final approval of proofs. We confirm that we have provided a current, correct email address which is accessible by the Corresponding Author.
